# Urinary Incontinence in Men with Stroke: A Cross-Sectional Study

**DOI:** 10.3390/medicina61010052

**Published:** 2025-01-01

**Authors:** İsmail Uysal, Özgür Nadiye Doğrukök, Yalcin Golcuk, Fatih Özden, Mehmet Özkeskin, Miray Başer, Bircan Yücekaya, Zeynep Nisa Karakoyun

**Affiliations:** 1 Department of Health Care Services, Fethiye Vocational School of Health Services, Muğla Sıtkı Koçman University, 48000 Muğla, Türkiye; ismailuysal@mu.edu.tr; 2 Physical Therapy Unit, Muğla Training and Research Hospital, Muğla Sıtkı Koçman University, 48000 Muğla, Türkiye; ozgurnadiye@gmail.com; 3 Department of Emergency Medicine, Faculty of Medicine, Muğla Sıtkı Koçman University, 48000 Muğla, Türkiye; yalcingolcuk@mu.edu.tr; 4 Department of Health Care Services, Köyceğiz Vocational School of Health Services, Muğla Sıtkı Koçman University, 48000 Muğla, Türkiye; 5 Department of Physiotherapy and Rehabilitation, Faculty of Health Sciences, Ege University, 35100 İzmir, Türkiye; mehmet.ozkeskin76@gmail.com; 6 Department of Physiotherapy and Rehabilitation, Institute of Health Sciences, Ege University, 35100 İzmir, Türkiye; miraybaser1@gmail.com; 7 Department of Physiotherapy and Rehabilitation, Faculty of Health Sciences, Ondokuz Mayıs University, 55200 Samsun, Türkiye; bircan.yucekaya@omu.edu.tr; 8 Department of Anatomy, Faculty of Medicine, Muğla Sıtkı Koçman University, 48000 Muğla, Türkiye; zeynepnisakarakoyun@mu.edu.tr

**Keywords:** bladder function, hemiplegia, male, physical function, life satisfaction

## Abstract

*Background and Objectives*: To evaluate urinary incontinence (UI) and its effect on quality of life (QoL) in male stroke patients. *Materials and Methods*: A quantitative cross-sectional study was conducted with 103 adult male stroke survivors. The individuals’ degree of disability was evaluated using the Modified Rankin Scale (MRS) and Barthel Index (BI). The UI assessment was performed with the Urogenital Distress Inventory (UDI-6) and the Incontinence Impact Questionnaire-7 (IIQ-7). In addition, the QoL was questioned with EuroQoL 5-Dimension 3-Level (EQ-5D-3L)”. *Results*: The mean age of the participants was 68.4 ± 9.9 years. The average scores of the IIQ-7 and UDI-6 were 9.7 ± 7.2 and 36.6 ± 26.3, respectively. According to these scores, both UI questionnaire results were slightly above the reference cut-off value. According to the UDI-6 scores, 52.4% of the participants exhibited symptoms, while 55.3% demonstrated symptoms according to the IIQ-7 scores. The IIQ-7 was strongly correlated with the MRS (*p* < 0.001, r = 0.740), BI (*p* < 0.001, r = −0.770), EQ-5D-3L Index (*p* < 0.001, r = −0.804), and EQ-5D-3L VAS (*p* < 0.001, r = −0.679) scores. In addition, the UDI-6 was strongly correlated with the MRS (*p* < 0.001, r = 0.697), BI (*p* < 0.001, r = −0.730), EQ-5D-3L Index (*p* < 0.001, r = −0.726), and EQ-5D-3L VAS (*p* < 0.001, r = −0.623) scores. Furthermore, the IIQ-7 and UDI-6 scores were statistically higher in patients with cortical-level involvement (*p* < 0.05). Regression results showed that the IIQ-7 was associated with the MRS, BI, EQ-5D-3L Index, and EQ-5D-3L VAS scores (R^2^ = 0.627, *p* < 0.001). Similarly, the UDI-6 was significantly associated with the MRS, BI, EQ-5D-3L Index, and EQ-5D-3L VAS scores in a multiple hierarchical regression model (R^2^ = 0.423, *p* < 0.001). *Conclusions*: The severity of UI was classified as high. As expected, UI was higher in individuals with increased disability. The QoL of individuals with UI is more negatively affected. Finally, the severity of UI was higher in individuals with cortical stroke.

## 1. Introduction

Urinary incontinence (UI), defined as the accidental or involuntary leaking of urine, is prevalent following a stroke. A stroke is a brain injury caused by vascular or circulation disorders in the brain [1]. More than half of post-stroke cases experience UI in the first month, 38% continue to have UI after one year, and 17% continue to have longer-term UI [2,3]. Incontinence is generally associated with poor stroke outcomes, depression, lower quality of life (QoL), social isolation, institutionalization, caregiver burden, and substantial economic burden [4]. As stroke patients’ control mechanisms, functionality, and independence decrease, UI problems occur, and this situation increases the risk of urinary tract infection by disrupting skin integrity and significantly affects the patient’s sleep, daily activities, self-confidence, and QoL [5].

In patients with a history of stroke, UI may manifest in two distinct ways: as voiding dysfunction or as an overactive bladder. However, UI is frequently associated with neurogenic overactive bladder, which in turn is linked to detrusor overactivity resulting from a loss of regulation of the micturition reflex [6]. Nocturia related to UI, especially when the bladder reaches its maximum level of arousal, is reasoned by conditions such as “frequency and urgency” [7]. The “pontine micturition center and the periaqueductal gray areas” interact to regulate the “micturition reflex”. The pontine micturition center exerts an excitatory influence, whereas the periaqueductal gray areas regulate the storage of urine in the bladder and act as an inhibitor [8]. “Periaqueductal gray areas” provide voluntary control of urine in conjunction with the limbic system and prefrontal cortex. Dysfunctions in the “prefrontal cortex and associated areas” adversely affect the regulation of the micturition reflex, resulting in an overactive bladder [9].

Post-stroke UI carries a poor prognosis for patients and caregivers, and ongoing UI is known to increase the physical and social burden on caregivers [10]. Since the aim of the assessment and rehabilitation program planned after stroke is to provide maximum functionality and independence and to restore QoL and activities of daily living, it is important to determine the impact of UI on patients. The QoL is severely impaired in both stroke patients and their caregivers due to UI, and many patients are unable to maintain their social, physical, personal, and sexual lives [11]. When neurological and motor deficits are combined with loss of motor control, the situation becomes much more serious for the patient and carer in terms of incontinence [12,13]. The role of UI, which is frequently investigated in women in the literature, is probably more complex and poorly understood in individuals with stroke [14]. UI is a prevalent condition affecting both men and women. However, the underlying causes can vary considerably between the two sexes. This discrepancy can be largely attributed to the distinct anatomical and physiological characteristics of men and women. Additionally, the risk factors that lead to the development of UI differ between the sexes. To illustrate, pregnancy, childbirth, and menopause represent significant risk factors for the onset of UI in women [15]. The strain on the pelvic floor muscles during pregnancy and childbirth can result in a weakening of their function, which may lead to stress incontinence [16]. Furthermore, hormonal alterations associated with menopause can contribute to urge incontinence [17]. Conversely, the most prevalent causes of UI in men are prostate issues, nerve damage, and the weakening of the sphincter muscle [16,18,19].

It is therefore important to evaluate the UI in terms of its impact on QoL, with a particular focus on sex-based differences. In this direction, when the literature was examined, no article was found that examined the relationship between incontinence and activities of daily living, QoL, function, and independence levels in men in the chronic period after stroke. This study will help to fill the gap in this field. The aim of this study was to evaluate the effect of UI on health-related QoL in male individuals with stroke.

## 2. Materials and Methods

### 2.1. Study Design and Participants

The data of our quantitative, analytical, cross-sectional study were obtained from volunteer stroke patients who were followed up as outpatients and inpatients at Ege University Faculty of Medicine Hospital and met the inclusion criteria. A total of 103 male patients, diagnosed with a first-time stroke, aged 65 years or above, with imaging-confirmed diagnoses and consenting to participate in the study, were included in the investigation. The study excluded female patients, those with additional neurological diseases, visual problems, severe aphasia, a history of urogenital disorders, and those who had undergone catheterization. The Ege University ethics committee approved the study protocol (No: 24-3.1T/12). All participants and their primary caregivers gave their informed consent.

### 2.2. Sample Size

Post-hoc analysis was performed with G-Power 3. Considering the lowest correlation coefficient (0.62) and R^2^ value (0.38), the effect size was calculated as 0.61. Regarding the possibility of 0.05 alpha error and the current sample size (103), the power of the sample was 99%.

### 2.3. Outcome Measures

Socio-demographic information, stroke-related clinical features, personal history, and family history were recorded.

#### 2.3.1. Incontinence Impact Questionnaire-7 (IIQ-7)

The effect of UI on the patient’s QoL was evaluated with the 7-item “Incontinence Impact Questionnaire-Short Form”, the Turkish version of which is valid and reliable in UI patients. It is evaluated as a four-point Likert scale. In this questionnaire, higher scores indicate a more significant negative impact on QoL, while lower scores indicate less impact on QoL [20].

#### 2.3.2. Urogenital Distress Inventory (UDI-6)

The instrument comprises six items, each employing a Likert scale (from 0 = none to 3 = severe). The first two questions of the scale (1st and 2nd) include irritative symptoms, the 3rd and 4th questions include stress symptoms, and the 5th and 6th questions include obstructive or voiding difficulty symptoms. The minimum score that can be attained on the scale is 0, while the maximum score is 18. The scores are then converted into a percentage. A reduction in the UDI-6 score is indicative of an improvement in QoL [20].

#### 2.3.3. Modified Rankin Scale (MRS)

The MRS is a functional recovery scale used to determine the degree of disability or dependency after a stroke. This scale assesses the functional status of patients and is scored from 0 to 6. Zero is the highest and six is the lowest functional level.

#### 2.3.4. Barthel Index

The scale consists of 10 items that assess mobility such as eating, washing, self-care, dressing, controlling bowel movements, controlling urination, going to the toilet, transferring from bed to wheelchair, walking or being dependent on a wheelchair, and climbing stairs on a scale of 5 to 15 [21].

#### 2.3.5. EuroQol EQ-5D-3L

It was developed to assess health-related QoL. Self-care, anxiety/depression, movement, pain/discomfort, and usual activities are the main 5 dimensions of this scale. Each scale dimension consists of one item with three options: no problem, some problem, and major problem. In addition, there is a visual analog scale (VAS) where individuals mark their current health status on a thermometer-like scale between 0 and 100. Turkish validity and reliability was transcribed [22].

### 2.4. Statistical Analysis

The study data were analyzed using SPSS v27 for Mac software. Normal distribution was assessed with the Kolmogorov–Smirnov test performed on MRS, BI, EQ-5D-3L Index, EQ-5D-3L VAS, UDI-6, and IIQ-7 variables. The Kolmogorov–Smirnov test showed that *p* values were less than 0.001 for all parameters. Since it was determined that the correlation analysis was not suitable for normal distribution, the partial correlation coefficient was preferred. A correlation (r) coefficient greater than 0.50 between two continuous variables represented a strong correlation. In addition, the Mann–Whitney U test was used to test the statistical significance between two different subgroups. The statistical significance value (*p*) was accepted as 0.05. In determining the independent predictive parameters of the IIQ-7 and UDI-6, multivariate linear regression analysis was performed with age, BMI, and stroke duration as control variables. The statistical significance and predictive percentage of the model were determined through the regression models’ significance level (*p*) and R^2^ value, respectively. Unstandardized ß, standardized ß, 95% CI, Durbin–Watson’s coefficient, variance inflation factor (VIF), and tolerance scores were also presented.

## 3. Results

The mean age of the participants was 68.4 ± 9.9 years. The mean BMI of the participants (26.0 ± 3.5) indicates that the majority of the population is classified as overweight, according to the standard criteria. The mean duration after stroke was 2.8 ± 2.3 years. The majority of the participants (56.3%) had a cortically affected stroke. As indicated by UDI-6 scores, 52.4% and by IIQ-7 scores, 55.3% of the participants were identified as exhibiting symptoms. Further details of the physical and demographic data of the participants are presented in Table 1.

The mean scores of the IIQ-7 and UDI-6 were 9.7 ± 7.2 and 36.6 ± 26.3, respectively. According to these scores, both UI questionnaire results were slightly above the reference cut-off value. This result revealed that the severity and QoL of the participants’ UI were above the norm. When the mean IIQ-7, UDI-6, MRS, BI, EQ-5D-3L Index, and EQ-5D-3L VAS scores of the two groups (symptomatic and asymptomatic) were compared according to IIQ-7 and UDI-6 scores, it was observed that the *p*-value of all parameters was <0.001. Functioning level and QoL scores of individuals and groups are given in Table 2.

Table 3 includes the correlation coefficients representing the relationship between the participants’ UI involvement levels with their QoL and functionality. The IIQ-7 was strongly correlated with the MRS (*p* < 0.001, r = 0.720), BI (*p* < 0.001, r = −0.695), EQ-5D-3L Index (*p* < 0.001, r = −0.765), and EQ-5D-3L VAS (*p* < 0.001, r = −0.701) scores. In addition, the UDI-6 was strongly correlated with the MRS (*p* < 0.001, r = 0.688), BI (*p* < 0.001, r = −0.665), EQ-5D-3L Index (*p* < 0.001, r = −0.655), and EQ-5D-3L VAS (*p* < 0.001, r = −0.579) scores.

Table 4 shows the effect of some clinical parameters on UI. According to the results, both IIQ-7 and UDI-6 scores were statistically higher in male stroke patients with cortical-level involvement (*p* < 0.05). Nevertheless, the side of involvement and other chronic diseases were found to have no significant effect on UI.

The regression results for the IIQ-7 and UDI-6 are presented in Table 5 and Table 6, respectively. A regression model proved that the IIQ-7 was associated with the MRS, BI, EQ-5D-3L Index, and EQ-5D-3L VAS scores (R^2^ = 0.627, *p* < 0.001, Table 5). According to the results of this model, the two parameters that predicted the IIQ-7 the most were the EQ-5D-3L Index (standardized ß = −0.393, *p* = 0.007) and EQ-5D-3L (standardized ß = −0.345, *p* = 0.001). Similarly, the UDI-6 was significantly related to the MRS, BI, EQ-5D-3L Index, and EQ-5D-3L VAS scores in a multiple hierarchical regression model (R^2^ = 0.423, *p* < 0.001, Table 6). The parameter most associated with the UDI-6 was the MRS (standardized ß = −0.334, *p* = 0.015).

## 4. Discussion

The objective of this quantitative, analytical, cross-sectional study was to evaluate UI and its effect on QoL in men with chronic stroke. The study also analyzed the relationship between UI and activities of daily living, QoL, functional status, and independence levels. The results of our study indicate that the prevalence of UI is significantly higher in men who have experienced a stroke. As anticipated, the prevalence of UI was higher in individuals with increased disability. The QoL is more adversely affected in men with UI. Furthermore, the severity of UI was found to be higher in individuals who had experienced a cortical stroke.

The study cohort consisted of 103 participants with a mean age of 68.4 ± 9.9 years and a mean time since stroke of 2.8 ± 2.3 years. Considering these findings, it appears that the average age at stroke is gradually decreasing. A review of the current literature reveals a decrease in the incidence of stroke in older adults, while the risk of stroke in young adults is higher. One potential explanation for the observed decline in the incidence of stroke in older adults is the possibility that the age of onset is gradually shifting toward a younger demographic [23,24].

Stroke and its associated symptoms have a detrimental impact on QoL. One of the symptoms that contributes to this negative effect is UI. UI persists as a prevalent issue among individuals who have experienced a cerebrovascular accident (CVA), even in the long term [25]. The psychological impact of UI, which may result from a number of underlying causes, can have a detrimental effect on the QoL of stroke patients, placing a considerable burden on their carers [4,26,27]. A review of the literature on UI and QoL reveals a consistent finding: lower urinary tract symptoms and UI have a negative impact on QoL, irrespective of sex [28,29]. However, since the female sex is a risk factor for UI, this situation has not been subjected to detailed investigation in men [30,31]. The IIQ-7 and UDI-6 scores were found to exceed the established cut-off values in male individuals who had experienced a stroke. This finding lends further support to the existing literature indicating that the QoL of men with stroke who experience UI is negatively affected.

The correlations analyzed during the research revealed a strong positive correlation between the Modified Rankin Scale, the Urogenital Distress Inventory, and the Incontinence Impact Questionnaire. This finding lends support to the interpretation that an improvement in functional independence is associated with an enhancement in the QoL in stroke survivors. A review of the literature reveals a similar conclusion in many other publications [32,33,34,35,36,37]. Similarly, a robust inverse correlation was identified between Barthel index scores and both the Incontinence Impact Questionnaire and the Urogenital Distress Inventory. This correlation indicates that a rise in the degree of independence in daily living activities is linked to an improvement in the QoL for stroke survivors. Furthermore, a robust inverse correlation was identified between the general QoL, pain levels, and scores from the Incontinence Impact Questionnaire and Urogenital Distress Inventory. This indicates that an enhancement in overall QoL and a reduction in pain levels result in an improvement in QoL pertaining to the urogenital system. A review of the existing literature reveals a correlation between the presence of UI in stroke patients and a number of adverse outcomes. These include increased dependence on others for activities of daily living, a decline in health-related QoL, reduced participation in social and leisure activities, and a lower level of life satisfaction [5,38,39]. Furthermore, incontinence has been linked to impaired cognitive function, and it has been recommended that bladder dysfunction be given due consideration during the rehabilitation of stroke patients [5].

A review of the literature reveals considerable variation in the reasons for UI in individuals with neurological diseases, including stroke. The risk factors for UI in stroke patients include sociodemographic factors, low educational level, functional status, medications, comorbidities, type and localization of stroke, chronic cough, poor cognitive status, general aphasia, neglect, visual field defects, and somatic sensory deficits [1,40,41]. In our analysis of the impact of clinical parameters on UI, we observed that the QoL associated with urinary dysfunctions was diminished in male stroke patients with cortical-level involvement. It is our contention that this finding should be taken into account among the risk factors associated with UI. The reason why patients who have had a stroke with cortical level involvement are more severely affected by UI than those with subcortical level involvement may be that the cortical layer is responsible for a number of functions, including cognition, awareness, memory, perception, and thought [42,43,44]. As a result, cortical involvement can negatively affect awareness, perception, and cognition, leading to a decrease in bladder control. This predisposes to UI. However, in contrast with the findings of previous studies, no correlation was identified between the side of involvement and the presence of comorbidities or UI. The reason for this discrepancy may be attributed to a number of factors, including sex, rehabilitation program, medications used, and the types of comorbidities present. It would be beneficial for future studies to be planned in order to clarify this situation.

Our regression model results revealed that functional status and QoL were predictors of the severity of UI. The IIQ-7 was most predictive of QoL and the UDI-6 was most predictive of functional status. These results are expected outcomes in terms of clinical implications. When analyzed in detail, we can suggest that the UDI-6 is more representative of the functionality of the individual, and the IIQ-7 is more representative of the QoL. In summary, incontinence in male stroke survivors is an essential clinical condition that has a direct relationship with QoL and functionality. This study is the first to address the impact of UI on functioning and QoL in male stroke survivors from a regressive perspective. Future studies may address more predictors of UI based on objective clinical findings.

### Limitations

The current study has some limitations. The first limitation is that the male population with stroke was derived from a single center. However, the relatively high number of participants serves to offset this disadvantage. A second limitation is that the presence of other chronic diseases was recorded, but no analysis specific to other chronic diseases was conducted. This is due to the fact that the diseases in question were not recorded in an explicit manner. Responses were recorded as either yes or no. This precluded a detailed analysis. A further limitation is that the various types of stroke were not recorded in accordance with the TOAST criteria.

## 5. Conclusions

The prevalence of UI in men with stroke is high enough to warrant consideration. It is therefore recommended that this be taken into account during the rehabilitation process. The presence of UI is associated with a reduction in independence and functionality, as well as a decline in health-related QoL. Moreover, UI is more prevalent among individuals who have experienced a cortical stroke. It is therefore recommended that cortical involvement be considered as a risk factor for UI.

## Figures and Tables

**Table 1:** The individual characteristics of men with stroke.

	Total (n = 103)
**Age (years, mean ± SD)**	68.4 ± 9.9
**BMI (kg/m^2^, mean ± SD)**	26.0 ± 3.5
**Duration after stroke (** **years, mean ± SD)**	2.8 ± 2.3
**Dominant side (right/left, n)**	91/12
**Affected side (right/left, n)**	64/39
**Stroke type (subcortical/cortical, n)**	45/58
**Marital status (single/married, n)**	18/85
**Education (university/high school/primary school, n)**	11/23/69
**Other chronic diseases (no/yes, n)**	28/75
**UDI-6 (symptomatic/asymptomatic)**	54/49
**IIQ-7 (symptomatic/asymptomatic)**	57/46

**n:** number, **SD:** standard deviation, **BMI:** body mass index.

**Table 2:** The average values of the clinical measurements.

	IIQ-7 Mean ± SD			UDI-6 Mean ± SD			Stroke Patients (n = 103) Mean ± SD
	Symptomatic (n = 57)	Asymptomatic (n = 46)	*p*	Symptomatic (n = 54)	Asymptomatic (n = 49)	*p*	
**IIQ-7**	15.31 ± 4.21	2.82 ± 3.04	<0.001	14.07 ± 5.92	4.95 ± 5.38	<0.001	9.7 ± 7.2
**UDI-6**	51.36 ± 24.08	18.35 ± 15.44	<0.001	57.09 ± 18.58	14.05 ± 10.27	<0.001	36.6 ± 26.3
**MRS**	2.87 ± 1.26	1.15 ± 1.09	<0.001	2.90 ± 1.20	1.22 ± 1.21	<0.001	2.1 ± 1.4
**BI**	63.87 ± 30.82	93.95 ± 14.00	<0.001	63.63 ± 31.79	92.38 ± 14.61	<0.001	77.3 ± 28.8
**EQ-5D-3L Index**	0.38 ± 0.34	0.83 ± 0.20	<0.001	0.38 ± 0.35	0.80 ± 0.22	<0.001	0.5 ± 0.3
**EQ-5D-3L VAS**	48.94 ± 21.47	74.56 ± 19.79	<0.001	48.98 ± 21.63	72.95 ± 20.71	<0.001	60.3 ± 24.2

**SD:** standard deviation, **n:** number of patients, ***p*:** *p* significance.

**Table 3:** The relationship between UI with QoL and functionality.

n: 103	IIQ-7	UDI-6
**MRS**	0.720	0.688
**BI**	−0.695	−0.665
**EQ-5D-3L Index**	−0.765	−0.655
**EQ-5D-3L VAS**	−0.70	−0.579

**n:** number of patients, partial correlation controlling for age, stroke duration, and BMI.

**Table 4:** The relationship between UI and QoL and disease-specific characteristics.

n: 103	IIQ-7	UDI-6
**Affected side**		
Right	10.1 ± 7.2	27.5 ± 3.4
Left	9.0 ± 7.2	23.9 ± 3.8
*p*-significance	0.424	0.185
**Stroke type**		
Subcortical	7.6 ± 5.2	26.0 ± 18.1
Cortical	11.3 ± 8.2	44.8 ± 28.8
*p*-significance	0.010	0.001
**Other chronic diseases**		
Yes	7.6 ± 7.0	33.7 ± 25.2
No	15.3 ± 7.2	37.7 ± 26.8
*p*-significance	0.069	0.499

**n:** number of patients.

**Table 5:** Multivariable linear regression analysis with IIQ-7 as the outcome (**R^2^ = 0.627, *p* < 0.001**).

n = 103					95% CI for Standardized ß	
		Unstandardized ß	Standardized ß	*p*	Lower Limit	Upper Limit
**Controlling variables**	**Age**	−0.115	−0.158	0.039	−0.224	−0.006
	**BMI**	0.003	0.001	0.984	−0.252	0.257
	**Stroke duration**	−0.288	−0.092	0.150	−0.682	0.106
**Independent variables**	**MRS**	1.161	0.235	0.063	−0.066	2.387
	**BI**	0.007	0.027	0.855	−0.066	0.079
	**EQ-5D-3L Index**	−7.801	−0.393	0.007	−13.402	−2.199
	**EQ-5D-3L VAS**	−0.103	−0.345	0.001	−0.162	−0.045

n: number of patients, MRS: Modified Rankin Scale, BI: Barthel Index, IIQ-7: Incontinence Impact Questionnaire-7 (IIQ-7), EQ-5D-3L: EuroQoL 5-Dimension 3-Level, VAS: Visual Analog Scales, Durbin–Watson’s coefficient = 1.64, variance inflation factor (VIF) < 5.99, and tolerance over = 0.96.

**Table 6:** Multivariable linear regression analysis with UDI-6 as the outcome (**R^2^ = 0.423, *p* < 0.001**).

n = 103					95% CI for Standardized ß	
		Unstandardized ß	Standardized ß	*p*	Lower Limit	Upper Limit
**Controlling variables**	**Age**	0.409	0.155	0.061	−0.018	0.836
	**BMI**	1.108	0.149	0.030	0.108	2.108
	**Stroke duration**	−2.171	−0.192	0.006	−3.715	−0.626
**Independent variables**	**MRS**	5.993	0.334	0.015	1.183	10.804
	**BI**	−0.170	−0.186	0.240	−0.454	0.115
	**EQ-5D-3L Index**	−4.334	−0.060	0.696	−26.307	17.639
	**EQ-5D-3L VAS**	−0.212	−0.195	0.069	−0.441	0.017

n: number of patients, MRS: Modified Rankin Scale, BI: Barthel Index, UDI-6: Urogenital Distress, EQ-5D-3L: EuroQoL 5-Dimension 3-Level, VAS: Visual Analog Scales, Durbin–Watson’s coefficient = 2.05, variance inflation factor (VIF) < 5.99, and tolerance over = 0.96.

## Data Availability

All data generated or analyzed during this study are included in this published article.
